# A Review on *Aspergillosis* in Turkey: As a Main Fungal Disease in Poultry

**DOI:** 10.1002/vms3.70605

**Published:** 2025-09-24

**Authors:** Ansam Naji Aboud Alhassani, Abdulrahman T. Ahmed, Gaurav Sanghvi, Subbulakshmi Ganesan, Hussein Riyadh Abdul Kareem Al‐Hetty, I. B. Sapaev, Abhayveer Singh, Puneet Sudan, Yasser Fakri Mustafa, Majid Gholami‐Ahangaran

**Affiliations:** ^1^ Pharmacology and Toxicology Department Al kitab University, College of Pharmacy Iraq; ^2^ College of Nursing University of Al Maarif Al Anbar Iraq; ^3^ Marwadi University Research Center Department of Microbiology Faculty of Science Marwadi University Rajkot Gujarat India; ^4^ Department of Chemistry and Biochemistry School of Sciences JAIN (Deemed to be University) Bangalore Karnataka India; ^5^ Department of Biology College of Education for Pure Sciences University of Anbar, Ramadi Anbar Iraq; ^6^ Head of the Department of Physics and Chemistry Tashkent Institute of Irrigation and Agricultural Mechanization Engineers National Research University Tashkent Uzbekistan; ^7^ Scientific Researcher University of Tashkent for Applied Sciences Tashkent Uzbekistan; ^8^ Western Caspian University Baku Azerbaijan; ^9^ Centre for Research Impact & Outcome Chitkara University Institute of Engineering and Technology Chitkara University Rajpura Punjab India; ^10^ Department of Pharmacy Chandigarh Pharmacy College Chandigarh Group of Colleges—Jhanjeri Mohali Punjab India; ^11^ Department of Pharmaceutical Chemistry College of Pharmacy University of Mosul Mosul Iraq; ^12^ Department of Clinical Sciences Faculty of Veterinary Medicine Shahrekord Branch Islamic Azad University Shahrekord Iran

**Keywords:** *aspergillosis*, brooder pneumonia, mycotic diseases, Turkey

## Abstract

**Background:**

*Aspergillosis*, a fungal disease caused by various species of the genus *Aspergillus*, poses a significant threat to the health and productivity of turkeys.

**Objective:**

The current review aims to synthesize current knowledge regarding the aetiology, pathogenesis, clinical manifestations, diagnostic methods and management strategies associated with *aspergillosis* in turkeys.

**Methods:**

A simple narrative literature review was conducted in Google Scholar, PubMed and ScienceDirect databases to identify relevant studies published in peer‐reviewed journals. The review focused on the pathogenesis of *aspergillosis* in turkeys, clinical manifestations, diagnostic techniques and therapeutic strategies.

**Results:**

*Aspergillosis* typically results from environmental exposure to spores, particularly in settings with poor ventilation and high temperature and humidity, leading to respiratory distress and systemic disease. Clinical signs can vary widely, ranging from mild respiratory symptoms to severe systemic illness, which complicates diagnosis due to overlap with other respiratory diseases. Diagnostic approaches include clinical evaluation, necropsy findings and laboratory tests such as fungal culture and molecular techniques. Effective management requires a multifaceted strategy that encompasses improved biosecurity measures, environmental control and appropriate treatment protocols.

**Conclusion:**

Understanding the epidemiology and impact of *aspergillosis* is crucial for developing effective preventive strategies and mitigating its economic consequences in turkey production. This review underscores the need for continued research to enhance diagnostic capabilities and therapeutic options, ultimately contributing to better health outcomes in turkey populations.

## Graphical abstract

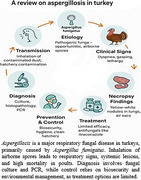

## 1 Introduction

Fungi, tiny organisms with chitin in their cell walls, can cause problems for turkeys in two main ways: directly through infection and indirectly through the production of mycotoxins (Gholami‐Ahangaran et al. 2016). Although fungi are common in the environment, infections rarely cause disease unless the turkey's immune system is weakened or its gut bacteria have been disrupted by antibiotics (Chege 2016). Broad‐spectrum antibiotics, although essential for treating bacterial infections, exert profound effects on the gut microbiome. They cause a loss of microbial diversity, especially in commensal anaerobic bacteria that play essential roles in maintaining mucosal immunity, metabolic homeostasis and competitive exclusion of pathogens (Gholami‐Ahangaran et al. 2022). Studies have shown that microbiota‐depleted mice have impaired alveolar macrophage function and neutrophil recruitment, which are both critical for controlling *Aspergillus* spores (Tejeda‐Garibay and Hoyer 2023).

The most economic important fungal infections in turkeys are *aspergillosis* and *candidiasis* (Arné and Lee 2020). *Aspergillosis*, also known as brooder pneumonia, is primarily a respiratory infection caused by *Aspergillus* species, primarily *A. fumigatus* and *A. flavus*. This disease is not contagious, but it can cause serious problems, especially in young poults (Vahsen et al. 2021).

*Aspergillus* spores are ubiquitous in the environment, but high concentrations in places like dusty hay, compost or litter can lead to infection. Spores, tiny enough to reach the deep lungs, can cause inflammation and tissue damage. The infection can also spread to other organs, such as the liver, brain and joints (Shaapan and Girh 2024).

*Aspergillosis* is mainly characterized by symptoms affecting the respiratory, neurological, visual and musculoskeletal systems. Respiratory distress includes challenges with breathing, gasping and quick breathing. The neurological symptoms comprised torticollis (head tilt) and lack of coordination (Seyedmousavi et al. 2015). Eye issues, such as cloudiness, conjunctivitis and keratitis, may occur in *aspergillosis*. Additionally, lameness may be observed due to inflammation in the hip joints or compression of the spinal cord (Shivaprasad 2014).

*Aspergillosis* is a significant health concern in turkeys. Although *Aspergillus* species are ubiquitous in the environment, they can cause various health problems in turkeys, including respiratory distress, mycotic omphalitis, and even systemic infections (Munir et al. 2017).

*Aspergilosis* can be diagnosed by isolating the fungus or finding fungal hyphae in samples (Vahsen et al. 2021). However, there are no approved antifungal drugs for food‐producing animals. Prevention focuses on avoiding immunosuppression and reducing exposure to spores through good hygiene practices (Meade et al. 2021).

The fungal challenge highlights the importance of maintaining a strong immune system in turkeys and reducing exposure to fungal spores and mycotoxins through good husbandry practices (Arné and Lee 2020).

## 2 Aetiology and Pathogenesis

There are many different types of *Aspergillus* species, but most cases of *aspergillosis* in turkey (*Meleagris gallopavo*) are caused by *A. fumigatus* and/or *A. flavus* (Arastehfar et al. 2021). *Aspergillus* species have been found in the brains of turkey poults and can also affect chickens and game fowl (Shaapan and Girh 2024). These fungi grow into hyphae and produce spores called conidia, which can be found in various environmental substrates like hay or compost. Conidia can become airborne when disturbed and are inhaled by hosts, causing infection and clinical symptoms, particularly in young turkeys (Vahsen et al. 2021).

Factors like stress, poor nutrition or virus infections can weaken the immune system and make birds more susceptible to *aspergillosis*. Some strains of *A. fumigatus* have been found to be more virulent than others, but there is no clear host‐specificity among these strains (Asfaw and Dawit 2017). *Aspergillus* species produce enzymes that can damage host tissues, leading to inflammation and tissue destruction. The role of mycotoxins produced during infection in disease pathogenesis is still debated, with some toxins like gliotoxin showing increased levels in infected turkeys (Hafez and Shehata 2021).

*Aspergillus* species can penetrate eggshells, leading to potential infection of the embryo, resulting in either death or hatching with a fully grown lesion (Shaapan and Girh 2024).

Aflatoxin is generated during the infection stages of *A. flavus* and *A*. *fumigatus* (Ghazaei 2017). However, the increased pathogenicity of aflatoxin‐producing strains in turkey poults has not been conclusively demonstrated. Infections with conidia from *A. flavus* do not elicit an antibody response or result in mortality among turkey poults. In contrast, aerosol infection with *A. fumigatus* is associated with a 50% mortality rate and the production of antibodies in these birds (Shahabi‐Ghahfarokhi et al., 2016). Clinical symptoms, including neck deviation in turkeys without central nervous system lesions (Crespo et al. 2018), suggest that the toxin produced by *A. fumigatus* may contribute to the manifestation of these symptoms (Guzman 2015).

Gliotoxin is one of the toxins produced by *A. fumigatus* (Scharf et al. 2012). Turkeys exhibit sensitivity to oral exposure to this toxin, which is known to suppress the immune system and exhibit cytotoxic effects. Specifically, gliotoxin inhibits blastogenesis in the peripheral blood lymphocytes of turkey poults (Li 2014). Notably, elevated levels of this toxin have been detected in the lungs of infected turkeys (Richard and DeBey 1995).

## 3 Influence of Environmental and Management Factors

*Aspergillosis* in turkeys is strongly influenced by environmental conditions and farm management practices that affect the proliferation and airborne dissemination of *A. fumigatus* spores. Understanding these factors is critical for effective prevention (Seyedmousavi et al. 2018).

Environmental factors such as temperature and humidity play a key role. *Aspergillus* thrives in warm (25–37°C), humid (>70% relative humidity) environments commonly found in poorly ventilated poultry houses (Shaapan and Girh 2024). Moisture accumulation in litter, feed and housing materials provides an ideal substrate for fungal growth. Dust particles also carry spores through the air, increasing respiratory exposure risk (Lacey 2024).

Management practices significantly affect spore loads and disease risk. Hatchery contamination due to inadequate sanitation and ventilation is a major source of early‐life infection. Similarly, improper brooding conditions—such as excessive humidity, poor ventilation and inappropriate temperatures—promote fungal growth and suppress poults’ immune defences. Feed storage under humid conditions encourages mould growth, which increases both fungal exposure and immune suppression in birds (Asfaw and Dawit 2017).

Effective control measures focus on reducing environmental spore loads and minimizing bird exposure. These include maintaining dry, regularly replaced litter and ensuring good ventilation to keep humidity below 60%. Dust control through misting and cleaning ventilation systems reduces airborne spores. Hatchery sanitation with thorough cleaning, disinfection and air filtration is crucial. Proper feed storage in dry, ventilated areas prevents mould contamination. Although antifungal treatments in bedding and prophylactic supplements like probiotics show promise, further research is needed (Prasad 2019; Collett et al. 2020).

## 4 Incubation Period

In turkey poults, mortality rates of 75% began at 5 days, primarily due to acute aspergillosis infection. Beyond 7 days, aflatoxin exposure exacerbates immunosuppression, which likely contributes to the peak mortality observed at 15 days, before rates decline by 3 weeks (Kuncle 2003). Some affected poults exhibited convulsive symptoms and died within 24 h. Notably, clinical symptoms of the disease in experimental *aspergillosis* do not become apparent until 48 h after the challenge with a significant dose of *A. fumigatus* or *A. flavus* (Latgé and Chamilos 2019).

## 5 Prevalence and Mortality

*Aspergillosis* in young chickens and turkeys is associated with high mortality and morbidity, whereas adult poultry typically experience lower rates (Shaapan and Girh 2024). *Aspergillosis* exhibits higher mortality and morbidity in young poults compared to adult poultry primarily due to the immaturity of their immune system, which limits their ability to effectively combat fungal infections (Vahsen et al. 2021). Additionally, the anatomical and physiological development of the respiratory tract in poults is less advanced, making them more susceptible to fungal colonization and dissemination. In contrast, adults possess a more robust immune response and better respiratory defences, contributing to lower disease severity (Maina 2023).

In some flocks, the disease is identified only at the slaughterhouse, often in conjunction with lung lesions. The removal of turkey carcasses due to air sac inflammation is the second leading cause of carcass rejection in the United States (Jennison 2021). Outbreaks of *aspergillosis* have resulted in the loss of one‐third of affected turkey flocks, primarily due to litter contamination (Gomes et al. 2022). Modifying feed sources and litter can help mitigate infection (Rizwan et al. 2023). Approximately 50% of turkeys exposed to *A. fumigatus* died within 10 min, with 5 × 10^5^ colony‐forming units detected per gram of lung tissue (Cheng et al. 2020). In another study, turkey poults exposed to conidia of *A. flavus* did not experience mortality, possibly because the larger size of the conidia (up to 6 µm) compared to *A. fumigatus* spores (2–3 µm) limited their ability to penetrate the deeper regions of the respiratory tract. Conversely, all turkeys exposed to 2.2 × 10^6^ live units of *A. fumigatus* via aerosol administration died within 5 days, whereas a lower exposure level (5.2 × 10^5^ live units) resulted in delayed and reduced mortality, with losses beginning 3 to 4 days post‐exposure (Asfaw and Dawit 2017).

## 6 Clinical Sign

Clinical signs of *aspergillosis* in birds typically appear 2 days after infection, with turkeys more susceptible than chickens but less so than other bird species (Vahsen et al. 2021). The more susceptibility of turkeys can be related to differences in their respiratory anatomy and immune defences. Turkeys have less efficient mucociliary clearance and a comparatively weaker innate immune response, which facilitates fungal colonization and disease progression. Additionally, management and environmental stressors may exacerbate their vulnerability to *Aspergillus* infections (Maina 2023).

The disease can range from acute in young poults to chronic in mature turkeys, with varying levels of morbidity and mortality. Basic symptoms include lethargy (Timurkaan et al. 2017), weight loss (Kalkayeva et al. 2023) and increased thirst, whereas signs can be specific based on the affected organs (Vahsen et al. 2021). Severe cases may lead to death, with mortality rates as high as 75% reported (Timurkaan et al. 2017).

Lung *aspergillosis* is the most prevalent form of the disease in chickens, turkeys and adult breeding turkeys. Ophthalmitis, or inflammation of the eye, may occur, typically in turkeys with lung involvement, indicating haematogenous spread of the infection. Although dermatitis and lameness have been observed in other avian species, these symptoms have not been reported in turkeys (Arné and Lee 2020).

Two forms of ocular *aspergillosis* have been observed in poultry. In one form, the conjunctiva and the external surface of the eye exhibit cheesy exudate, with plaques visible beneath the third eyelid. This condition may result from contact between the conjunctival surface and a contaminated environment. The second form, predominantly seen in turkeys, is associated with respiratory *aspergillosis*. In this instance, the majority of the posterior chamber and the vitreous body of the eye are affected, likely due to haematogenous spread of the fungus (Shaapan and Girh 2024).

Mortality associated with ocular surface infections in hatcheries tends to increase following ophthalmo‐nasal vaccination against Newcastle disease. This type of eye involvement has been reported in five flocks of turkey poults and three breeder flocks (Arné and Lee 2020).

*A. fumigatus* infection in bone can lead to deformities of the vertebral column and paralysis in turkey poults. This condition likely arises following a pulmonary infection and subsequent haematogenous dissemination of the fungus. In a report from Germany, *A. fumigatus* was isolated from 7‐ to 11‐week‐old turkeys exhibiting symptoms of lameness, granulomatous osteoarthritis in the hock joint and necrosis of the femoral head (Dykstra et al. 2013).

Numerous reports document encephalitis or meningoencephalitis caused by *Aspergillus* in various bird species. Necrotic lesions in the brain and cerebellum have been observed in turkeys with naturally occurring *aspergillosis* (Seyedmousavi et al. 2015) and in turkey poults experimentally exposed to aerosolized conidia of *A. fumigatus*. Additionally, there is a report of encephalitis in turkey poults characterized by caseous necrotic lesions in the brain and cerebellum, as well as granulomatous encephalitis (Carrasco et al. 2016). Fungal hyphae have been confirmed in the cerebellum of turkeys exhibiting neurological symptoms. Isolates of *A. fumigatus*, *A. niger*, and *Penicillium variotii* have been obtained from internal organs in pneumomycosis associated with neurological symptoms (Brown et al. 2008). Furthermore, *A. fumigatus* and later *Diplodia* species have been isolated from turkey brains displaying symptoms of imbalance. In many instances, lung and air sac infections occur concurrently with kidney and liver involvement (Munkvold et al. 2019). Simultaneous lung and yolk sac infection with *A. fumigatus* has been reported in turkey poults (Munir et al. 2017).

Overall, the severity of clinical signs and outcomes depends on various factors such as age and the extent of infection.

## 7 Macroscopic Lesions

*Aspergillosis* in turkey manifests as small, white or opaque nodules in the air sacs and lungs. As the disease progresses, these nodules can transform into greenish‐grey mould, indicating the formation of conidia, which are fungal spores. In severe cases, the trachea can be obstructed by caseous plaques, and systemic *aspergillosis* can lead to lesions in various organs, including the intestine, liver, adrenal glands, kidneys, sternal bone, vertebrae and gizzard (Shaapan and Girh 2024). Brain lesions are characterized by necrotic, white‐to‐yellow, demarcated areas. Mycotic omphalitis, a form of infection in the navel, results in pasty or watery, greenish to yellow‐brown yolk contents and prominent, red navels (Arulmozhi et al. 2016).

Systemic *aspergillosis* caused by *A. flavus* in turkeys can affect the sternum (Shaapan and Girh 2024). Intravenous inoculation of conidia from *Aspergillus* leads to acute millet hepatitis (Navale et al. 2021).

Uncomplicated pulmonary *aspergillosis* lesions can develop within days and may resolve after several weeks. In acute experimental *aspergillosis* in turkeys, the lesions can quickly become undetectable. Within 24 h, white nodular lesions form on the walls of the air sacs, accompanied by red gelatinous oedema in the lungs. As the disease progresses, the air sacs thicken and darken, leading to the formation of granulomatous nodules (Shaapan and Grih 2024). In experimental aerosol infections, cheesy‐white pulmonary lesions, approximately 10 mm in size, are typically observed throughout the lungs, often with caseous plaques in the air sacs. Bloody ascites may also be present. Yellow‐to‐white nodules have been noted in the lungs of wild turkeys raised in cages (Mangus et al. 2021). In one instance of *aspergillosis*, no white nodules were found in the lung; instead, the lung appeared grey‐yellow. The first report of omphalitis in 5‐ to 9‐day‐old turkeys in the United States involved the isolation of *A. fumigatus* from the yolk sac, which was associated with yellow fungal nodules in the lungs (Arné and Lee 2020). This isolation of *A. fumigatus* from the yolk sac in young poults suggests a possible route of vertical transmission, where the fungus may be transmitted from the breeder hen to the embryo before or during incubation. Vertical transmission could contribute to early‐onset aspergillosis, highlighting the need for further investigation into fungal contamination of eggs and breeder flocks (Shaapan and Grih 2024).

In advanced *aspergillosis*, when the organism is transmitted through the air, it can sporulate and produce green‐to‐grey fungal growth on the surface of cheesy lesions and the walls of the air sacs (Rizwan et al. 2023). Lesions in the brain typically present as yellow‐to‐white spots that are often visible on the surface. These lesions may be located in the brain or cerebellum and, although rare, can occur in both areas (Hernández 2014).

## 8 Immunity

There is currently no evidence of immunity against *aspergillosis* in poultry. However, most turkeys that survive an experimental infection with *A. fumigatus* after 4–5 weeks of recovery are not re‐infected (Vahsen et al. 2021). Recovery from pulmonary *aspergillosis* has also been reported in Japanese quail (Abd El‐Ghany 2019), although the mechanisms of recovery in various bird species have not been documented. In immunocompromised mammals with pulmonary *aspergillosis*, an independent macrophage and neutrophil response has been observed to clear lung tissue (Mirkov et al. 2019).

## 9 Diagnosis

### 9.1 Histopathological Observations

Histopathological examinations reveal that *A. fumigatus* and *A. flavus* cause similar lesions. Early lesions include thickened air sac membranes with oedema and an influx of inflammatory cells like heterophils and macrophages. Necrotic epithelium can be observed, leading to erosions covered by exudate. Focal lesions are characterized by clusters of epithelioid macrophages, multinucleated giant cells and granulomas, some with central necrosis (Jezdimirović et al. 2019). Lung lesions are primarily located in the pleura and adjacent tissue, resembling those in the air sacs. In advanced stages, pyogranulomas with a necrotic core develop, surrounded by intact heterophils and demarcated by epithelioid macrophages and fibrous tissue (Ozmen and Dorrestein 2004).

Turkeys that were experimentally exposed to aerosolized fungal spores exhibited cloudy eyes with inflammation of the retina, choroid, and iris (Shivaprasad et al. 2022). Histopathological examination revealed infiltration of heterophils and macrophages, along with cellular debris and fungal components in the anterior chamber and retina. The pecten is also significantly affected, showing oedema, heterophil infiltration, mononuclear cell accumulation and the presence of fungal components. In some cases, the pecten develops granulomatous lesions (Jezdimirović et al. 2019).

### 9.2 Isolation

**FIGURE 1:**
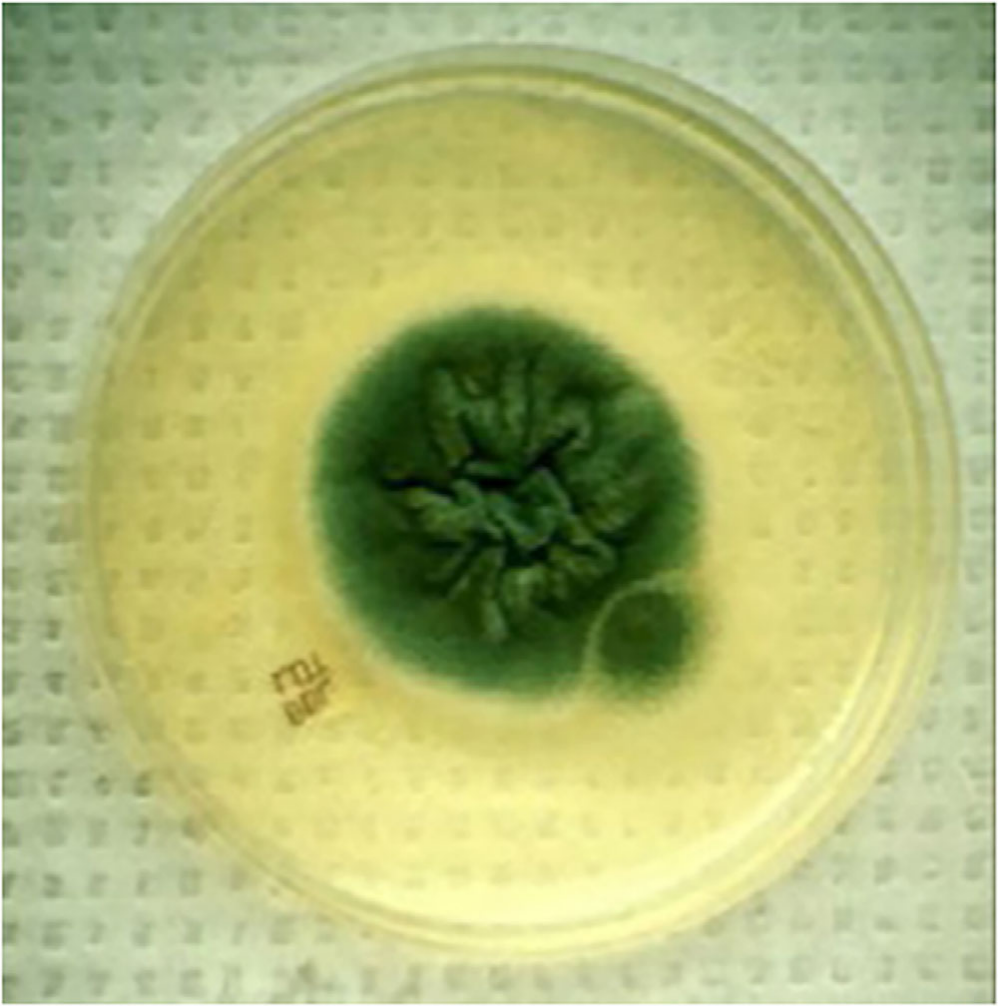
*A. fumigatus on* Sabouraud dextrose agar. *Source*: Courtesy of Dr. H. L. Shivaprasad.

Detection of *Aspergillus* spp. is done by directly examining samples under a microscope following treatment with 20% KOH. This permits the recognition of septate hyphae in the exudate. Proper sample collection is essential for isolating *Aspergillus* spp. because they are found everywhere (Romero et al. 2021). These fungi easily thrive in different laboratory substances, with Sabouraud dextrose agar being the most popular choice. Additional acceptable options are Czapek's solution and potato dextrose agar. *A. fumigatus* colonies have a blue–green centre *and A. flavus* colonies have a yellow–green‐to‐olive green centre with brown pigment (Shtayeh 1998) (Figure 1).

### 9.3 Molecular Detection

Molecular methods are useful for identifying *Aspergillus* spp. PCR with primers that are universal and target the internal transcribed spacer (ITS) region (Fagbohun et al. 2021). The 8S rRNA gene, ITS‐2 region or a part of the large subunit rRNA gene is often used to identify *Aspergillus* species (Arbefeville et al. 2017). PCR analysis can be conducted on DNA taken from isolated fungi or tissue that is embedded in paraffin. Real‐time PCR (qPCR) offers a precise way to measure the number of fungi present in the lungs (Vergidis et al. 2020). Nanofluidic PCR platforms are capable of identifying a range of avian respiratory pathogens, such as *A. fumigatus*. Multiple‐locus variable‐number tandem repeat analysis and microsatellite typing play a valuable role in epidemiological studies (Xie et al. 2022; Imbert et al. 2023).

## 10 Serological Examination

Serological methods for detecting antibodies against *Aspergillus* spp. are not commonly used in routine diagnostics and are rarely employed in research. Although agar gel precipitation tests (AGPT) have shown potential in detecting antibodies against *A. fumigatus*, results can vary depending on the isolate's ability to induce antibody production (Hauck 2024). Enzyme‐linked immunosorbent assays (ELISAs) have shown some correlation with AGPT results but are also prone to variability. Field studies using AGPT and passive haemagglutination tests have indicated low specificity for detecting *A. fumigatus* antibodies. Antigen detection using ELISA systems with commercially available *Aspergillus* antigen has shown promise, with increased antibody titres observed in some turkey flocks with *aspergillosis*. However, antigen detection lacks species specificity (Pereira et al. 2020).

The use of serological methods to detect *aspergillosis* in turkeys may be the preferred approach for identifying infected birds, as there is no effective treatment available for those that test positive (Hauck et al. 2020).

## 11 Prevention

Prevention is the most critical strategy for *aspergillosis* control. Prevention of *aspergillosis* relies on good management practices to minimize immunosuppression and infection pressure. Improved ventilation can reduce airborne fungal levels. Anecdotal evidence suggests that adjustments in ventilation to minimize dust, elimination of mouldy feed and better litter management can dramatically decrease the prevalence of *aspergillosis* (Rashidi et al. 2020).

Effective prevention typically involves the removal of contaminated sources, such as litter and feed, as well as the disinfection of poultry houses and litter using antifungal compounds (Corrêa‐Junior et al. 2024). Despite these precautionary measures, *aspergillosis* can still occur in some poultry houses, particularly during specific times of the year, such as winter, and in closed breeding facilities. Increasing ventilation in poultry houses has been shown to reduce air contamination (Witkowska and Sowińska 2017) and is recommended as an effective method for preventing *aspergillosis*. Natural ventilation is preferred over forced air ventilation; however, the impact of design and implementation of natural ventilation on turkey performance parameters—such as mortality rates, average daily weight gain, feed conversion ratios, carcass removal rates at the slaughterhouse and average body weight—has not been conclusively demonstrated (Munir et al. 2017).

Thoroughly cleaning turkey houses, drinkers and feeders, as well as hatcheries, helps eliminate potential breeding grounds for moulds and fungi. Although *Aspergillus* spp. are resistant to many chemical agents, phenolic compounds are effective disinfectants (Mattei et al. 2013). Litter treatments with nystatin, copper sulphate or thiabendazole have successfully reduced mortalities or pulmonary lesions in turkeys (Arné and Lee 2020). Mortality due to *aspergillosis* on farms is significantly reduced following 2 days of litter treatment with an enilconazole solution (Seyedmousavi et al. 2018). Additionally, essential oils with antimycotic activity against *A. fumigatus* may be effective for environmental treatment (Wang et al. 2022).

## 12 Treatment

Treatment of *aspergillosis* in turkeys is limited to preventing predisposing factors and providing supportive care. Although some azole compounds, particularly itraconazole, have shown efficacy against experimental infection, no antimycotic drugs are currently registered for use in food‐producing animals (Hassan et al. 2020).

There is generally no effective treatment for avian *aspergillosis*. Although certain medications are available for treating *aspergillosis* in mammals, these drugs are not economically viable for use in poultry (Melo et al. 2020).

Infection in chicken embryos has been effectively controlled using amphotericin B (Tokarzewski et al. 2012) and phenylmercury diphenylmethane disulphonate. Subcutaneous injection of dimethyldithiocarbamate has proven effective against *A. fumigatus* infection in 5‐ to 10‐week‐old chickens, resulting in a significant reduction in lesions and the isolation of the organism from tissues when compared to untreated infected birds. Fumigation with enilconazole during experimental infection with *A. fumigatus* also reduced the rate of infection and mortality (Dykstra et al. 2013). In another experimental study comparing azole compounds for the treatment of turkeys via crop gavage, itraconazole emerged as the most effective drug for reducing lesions and preventing weight loss (Arné and Lee 2020). Additionally, miconazole has been successfully used to treat *aspergillosis* in game birds (Charlton et al. 2008).

A critical concern is the emergence of antifungal resistance in *A. fumigatus*, particularly to triazoles, which are commonly used as environmental disinfectants in poultry hatcheries (e.g., enilconazole, thiabendazole). Although therapeutic antifungals are not routinely administered to turkeys, sublethal environmental exposure to azoles selects for resistant fungal populations (Gisi 2022).

Resistance mechanisms are often driven by mutations in the cyp51A gene, which encodes the target enzyme 14α‐demethylase. Notably, tandem repeat mutations in the cyp51A promoter region (e.g., TR_34_/L98H, TR_46_/Y121F/T289A) result in overexpression and azole resistance, often across multiple triazoles. Additional non‐cyp51A mechanisms, including efflux pump overexpression and biofilm‐mediated tolerance, further complicate treatment. These resistant strains are increasingly being detected in environmental and clinical settings, highlighting a concerning overlap (Melo et al. 2020).

Although resistance has not yet been widely documented in avian isolates, Cabañes et al. emphasize the lack of surveillance in poultry environments, despite environmental conditions being conducive to resistance selection. This gap poses a potential zoonotic risk, as resistant strains may spread from farm environments to humans, particularly immunocompromised individuals (Cabañes 2021).

The rise of antifungal resistance in *A. fumigatus*, particularly in avian species like turkeys, has intensified the search for alternative therapeutic approaches. Recent studies emphasize the potential of immunomodulators, phytotherapeutics, experimental vaccines and avian‐specific antifungal agents—strategies that benefit from translational studies across species (Orimaye et al. 2024).

Adjunctive immunotherapy has shown promise in enhancing antifungal efficacy. Granulocyte/Macrophage colony‐stimulating factors (G‐/GM‐CSF) combined with amphotericin B or caspofungin improved survival rates by up to 78% in murine invasive aspergillosis (IA) models (Damiani et al. 2020). IFN‐γ has been used to restore Th1 responses in immunocompromised patients (e.g., bone marrow or kidney transplant recipients), enhancing IL‐17/IL‐22 production and fungal clearance (Wang et al. 2023).

Plant‐derived agents such as essential oils (e.g., oregano, thyme) contain active flavonoids and terpenoids with dual antifungal and immunostimulatory properties (Salako et al. 2022, 2024). Although most data derive from in vitro and rodent models, these agents offer promising eco‐friendly alternatives for poultry farming (Kumari et al. 2024). Species‐specific pharmacokinetics in birds warrant further investigation.

Studies indicate that turkeys mount a weaker CD8+ T‐cell and cytokine response to *A. fumigatus* compared to chickens, necessitating tailored antifungal therapies (Vahsen et al. 2021). Novel agents targeting calcineurin signalling or mitochondrial functions are under evaluation. Cross‐species pharmacodynamic and safety trials are crucial to validate efficacy in avian hosts while minimizing drug residues in food products (Yadav and Heitman 2023).

## 13 Vaccines

Commercially available vaccines against *aspergillosis* are not currently available. Experimental vaccination of turkeys against *aspergillosis* provides only limited protection, reducing mortality (Tell et al. 2019) and primary pathological lesions, but it does not prevent lung lesions and may increase the susceptibility to chronic infection (Asfaw et al. 2017). Some vaccinated birds remained culture‐positive for *A. fumigatus* 8 weeks after the challenge, whereas both control non‐vaccinated birds became culture‐negative (Femenia et al. 2007).

Infected turkeys do not seem to develop lasting immunity after infection clearance. Passive immunization with splenocytes from *A. fumigatus*–infected birds has also been unsuccessful (Desoubeaux and Cray 2018). Vaccination using inactivated germinated conidia or mycelium has shown some partial protection against mortality, lung lesions and fungal burden (Ogwuegbu and Mthiyane 2024). Furthermore, recovery from *aspergillosis* does not confer effective protection to turkeys; those that recover from unilateral *A. fumigatus* alveolitis are not protected against contralateral alveolar involvement (Desoubeaux and Cray 2018).

Recent vaccine candidates, such as VesiVax liposomal formulations containing Asp f3 and Asp f9, have conferred protection in neutropenic mice by enhancing antigen‐specific IgG2a and IL‐4 responses (Slarve et al. 2022). Kexin‐based subunit vaccines (Af. KEX1) significantly reduced fungal burden and mortality in murine pulmonary aspergillosis (Rayens et al. 2022). Additionally, an oral *E. coli*–based vaccine expressing α‑Gal epitopes stimulated protective anti‐α‐Gal antibodies in avian models (Mateos‐Hernández et al. 2020), suggesting feasibility for poultry application.

## 14 Immunological Particularities of Turkeys for Development of Targeted Vaccines and Therapies

Turkeys exhibit distinct immunological features compared to other avian species, particularly chickens, which are the most widely studied model in avian immunology. Understanding these differences is critical for designing effective vaccines and immunotherapies against diseases such as *aspergillosis*, a significant respiratory mycosis in turkeys with high morbidity and economic losses in commercial settings (Davison 2022).

The innate immune response is the first line of defence against *A. fumigatus* conidia. Turkeys possess a robust but distinct pattern recognition receptor (PRR) profile, including Toll‐like receptors (TLRs) and C‐type lectin receptors (CLRs), which are key in fungal recognition (Smith and Fiddaman 2022). For instance, turkey macrophages have demonstrated altered expression of TLR2 and TLR4 compared to chickens, potentially leading to differences in pro‐inflammatory cytokine release and phagocytosis efficacy (Barjesteh et al. 2014). Moreover, heterophils (the avian equivalent of neutrophils) in turkeys show variations in degranulation and oxidative burst responses, which could affect early fungal clearance (Vahsen et al. 2021).

In turkeys, the adaptive immune system is characterized by differences in the development and function of T and B lymphocytes. Studies have shown that turkey T cells exhibit a slower proliferative response to mitogens compared to chickens, suggesting differences in cell‐mediated immunity. This is particularly relevant for defence against intracellular stages of *Aspergillus* infection (Schat 2018). Furthermore, the turkey's immunoglobulin repertoire, especially IgY and IgA, shows altered kinetics and titres during mucosal responses, potentially impacting the effectiveness of humoral immunity at the respiratory interface (Härtle et al. 2022).

*Aspergillosis* primarily affects the respiratory tract, where mucosal immunity plays a critical role. Turkeys have fewer and structurally distinct bronchus‐associated lymphoid tissues (BALT) than chickens, which may compromise local immune surveillance and antigen presentation. The mucus composition and ciliary clearance rates in turkeys also differ, possibly contributing to a higher susceptibility to inhaled fungal spores (Pabst 2022).

Turkeys, particularly in the early post‐hatch period, have a delayed maturation of both innate and adaptive immune components compared to chickens. This immunological immaturity correlates with heightened susceptibility to aspergillosis in young poults. Understanding this developmental timeline is crucial for determining optimal windows for vaccine administration (Alkie et al. 2019).

The unique immunological landscape of turkeys necessitates species‐specific vaccine strategies. Subunit or vector‐based vaccines should consider PRR profiles and T‐helper cell polarization differences (Saylor et al. 2020). Mucosal vaccine delivery may be optimized by targeting the limited BALT and enhancing local IgA responses. Immunomodulators and adjuvants may need tailoring to overcome suboptimal T‐cell activation or macrophage responsiveness (Eshaghi et al. 2024).

In conclusion, a deeper understanding of turkey immunobiology is essential to designing effective prophylactic and therapeutic strategies against *aspergillosis*. Integrating these immunological insights into vaccine development will significantly improve disease control in commercial turkey production systems.

## 15 Economic Impact of *Aspergillosis* in Turkeys: Implications for Policy and Prevention

*Aspergillosis*, caused by *A. fumigatus*, is a significant fungal disease affecting turkeys, with notable economic consequences for producers and the broader poultry sector. The disease primarily impacts young poults, causing high mortality rates—often between 30% and 50% during outbreaks—resulting in immediate loss of stock and revenue (Shaapan and Grih 2024). Surviving birds commonly experience chronic respiratory problems, reduced growth rates and poor feed conversion efficiency, all of which reduce flock uniformity and market value. Additionally, affected carcasses may be downgraded or condemned at slaughter, further reducing profitability (George and George 2023).

Beyond direct production losses, *aspergillosis* increases operational costs. Veterinary expenses rise due to supportive treatments and management of secondary infections, whereas labour demands intensify for monitoring, treatment and sanitation. The disease often necessitates extended downtime between flocks for thorough cleaning and disinfection, leading to production delays and higher fixed costs (Rizwan et al. 2023).

At the sectoral level, *aspergillosis* outbreaks can disrupt supply chains by reducing the number of market‐ready turkeys, causing price instability and potential loss of consumer confidence. There may also be trade implications, especially if disease outbreaks lead to increased use of antibiotics and concerns over antimicrobial resistance (Dadgostar 2019).

Given these economic challenges, investing in preventive measures is critical. Improving hatchery and brooder hygiene, enhancing ventilation, developing turkey‐specific vaccines and implementing fungal surveillance can reduce the incidence and severity of *aspergillosis*. Policy frameworks that support research, producer education and financial incentives for biosecurity improvements can facilitate adoption of such measures (Jennison 2021).

## 16 Public Health

Fungal diseases have historically received limited attention in global health discourse. However, the burden of *aspergillosis* is rising in tandem with the growing population of immunosuppressed individuals, such as cancer patients, organ transplant recipients and those with HIV/AIDS. *Aspergillosis* now ranks among the leading causes of opportunistic fungal infections, with substantial morbidity and mortality (Walker 2017).

Globally, it is estimated that over 3 million people suffer from chronic pulmonary aspergillosis (CPA), with an annual incidence of IA exceeding 300,000 cases. The disease's burden is notably higher in low‐ and middle‐income countries (LMICs), where delayed diagnosis and limited access to antifungal therapy exacerbate outcomes (Tashiro et al. 2024). Moreover, the COVID‐19 pandemic has amplified attention to *aspergillosis*, as COVID‐19‐associated pulmonary aspergillosis (CAPA) has emerged as a critical complication in ICU patients, significantly affecting case fatality rates (Egger et al. 2022).

The nonspecific clinical presentation of *aspergillosis* often leads to underdiagnosis or misdiagnosis, particularly in resource‐limited settings lacking advanced diagnostic tools (Chakrabarti et al. 2023). Conventional diagnostics such as culture and histopathology are slow and insensitive, whereas serologic and molecular tests remain unavailable in many regions. Furthermore, antifungal resistance, especially to azoles—the frontline therapy against *Aspergillus* spp.—has increased due to agricultural azole use, creating an urgent need for integrated surveillance and stewardship programmes (Achilonu et al. 2024).

*Aspergillosis* exerts a considerable strain on healthcare resources. Treatment is prolonged and costly, involving hospitalization, imaging, laboratory diagnostics and long‐term antifungal therapy. Invasive forms, particularly IA, carry mortality rates of 30%–90%, depending on host immunity and timing of intervention. The indirect costs, including loss of productivity and prolonged morbidity, add to its public health significance (Kanaujia et al. 2023).

Despite its substantial burden, *aspergillosis* remains neglected in national and global health policies. Integrating fungal disease surveillance into existing infectious disease frameworks is essential. There is also a need for increased investment in diagnostics, antifungal research and healthcare worker training. Additionally, environmental and agricultural policies must be aligned to control azole‐resistant *Aspergillus* strains, linking fungal disease control to One Health approaches (Al‐Worafi 2024).

## 17 Conclusion

In conclusion, this review underscores the intricate interactions among environmental factors, host susceptibility and the pathogenicity of *Aspergillus* species. To mitigate the risks associated with this disease, effective management strategies are essential, including enhanced biosecurity measures, environmental controls and early detection protocols. Future research should prioritize elucidating the molecular mechanisms of infection and resistance, as well as developing targeted interventions to bolster the resilience of turkey populations against *aspergillosis*. By addressing these critical areas, we can better protect turkey health and promote sustainable production practices.

## Author Contributions

Majid Gholami‐Ahangaran and Ansam Naji Aboud Alhassani contributed to the design and construction of the idea for this review and also analysed the data and supervised the project. Majid Gholami‐Ahangaran, Abdulrahman T. Ahmed, Gaurav Sanghvi, Subbulakshmi Ganesan, Hussein Riyadh Abdul Kareem Al‐Hetty and I. B. Sapaev contributed to writing the body of the manuscript. Gaurav Sanghvi, Subbulakshmi Ganesan, Hussein Riyadh Abdul Kareem Al‐Hetty, I. B. Sapaev, Abhayveer Singh and Puneet Sudan contributed to the searching, classification and selection of suitable articles on this subject and prepared the literature review. Abhayveer Singh, Puneet Sudan, and Yasser Fakri Mustafa prepared the resources and drafted the primary version of the manuscript. Majid Gholami‐Ahangaran, Ansam Naji Aboud Alhassani and Abdulrahman T. Ahmed reviewed and edited the final version of the manuscript before submission.

## Ethics Statement

The authors have nothing to report.

## Conflicts of Interest

The authors declare no conflicts of interest.

## Peer Review

The peer review history for this article is available at https://www.webofscience.com/api/gateway/wos/peer‐review/10.1002/vms3.70605.

## Data Availability

All data are available and reserved near the corresponding author. The requests are answered.
